# Long-term result of hybrid procedure for an extensive thoracic aortic aneurysm in Takayasu arteritis: a case report

**DOI:** 10.1186/1749-8090-5-28

**Published:** 2010-04-20

**Authors:** Yukio Obitsu, Nobusato Koizumi, Naozumi Saiki, Satoshi Kawaguchi, Hiroshi Shigematsu

**Affiliations:** 1 Department of Vascular Surgery, Tokyo Medical University, 6-7-1 Nishishinjuku, Shinjuku-ku, Tokyo 160-0023, Japan

## Abstract

We herein present a 60 years old woman with Takayasu arteritis and an extensive thoracic aortic aneurysm who initially underwent a total aortic arch replacement. Then, in the second stage, thoracic endovascular aortic repair was performed using the elephant trunk graft as the proximal landing zone at four weeks after aortic arch repair. The postoperative course was relatively uncomplicated, but a type II endoleak was noted. Currently, about 5 years postoperatively, the slight type II endoleak from intercostal artery persists, but aneurism dilatation has not been noted, so the patient is being followed up.

## Background

Single-stage surgery is preferable for extensive thoracic aortic aneurysms, but because of the excessive invasiveness of this approach, staged surgery must sometimes be performed. When staged surgery is selected, rupture of the residual lesion during the interval period is always a concern, so the second stage of surgery must be scheduled as soon as possible after the first. We herein present a patient with Takayasu arteritis and an extensive thoracic aortic aneurysm who initially underwent a total aortic arch replacement. Then, in the second stage, thoracic endovascular aortic repair (**T**EVAR) was performed using the elephant trunk graft as the proximal landing zone. Long-term results have been satisfactory.

## Case presentation

**Figure 1:**
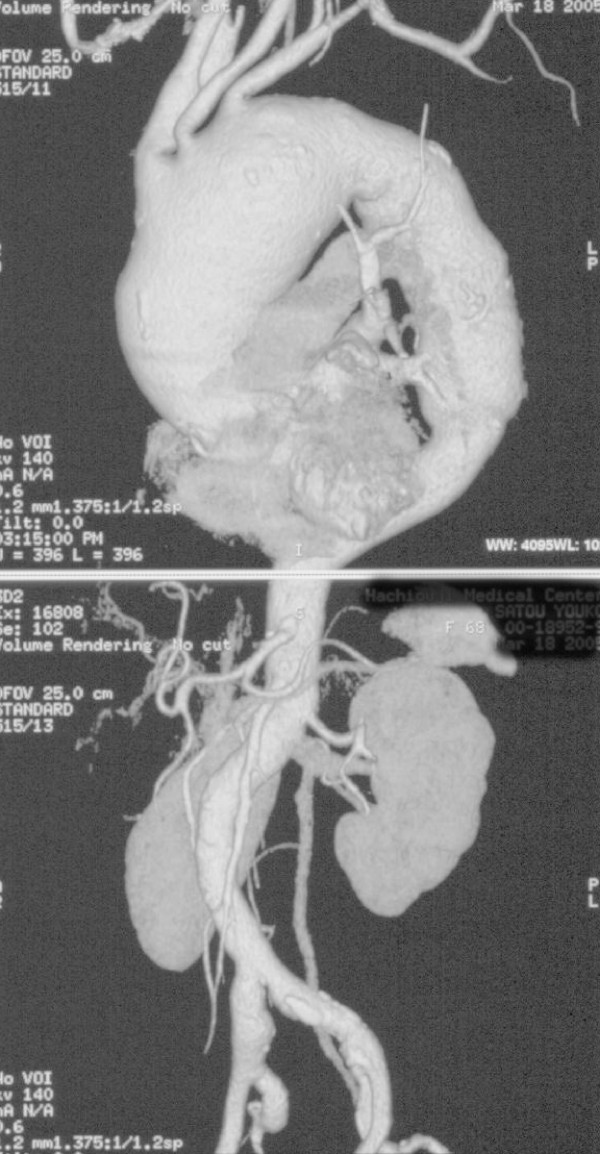
**3DCT scan obtained on admission, showing extensive thoracic aortic aneurysms**.

A 60-year-old woman who had been given a diagnosis of Takayasu arteritis about 20 years previously was being treated with steroids, but because of progressive dilatation of the ascending to mid-descending aorta, she was hospitalized for treatment (Figure 1). Further evaluation on admission revealed no complications, but because of susceptibility to infection due to long-term oral steroids, staged hybrid surgery with TEVAR was planned.

**Figure 2:**
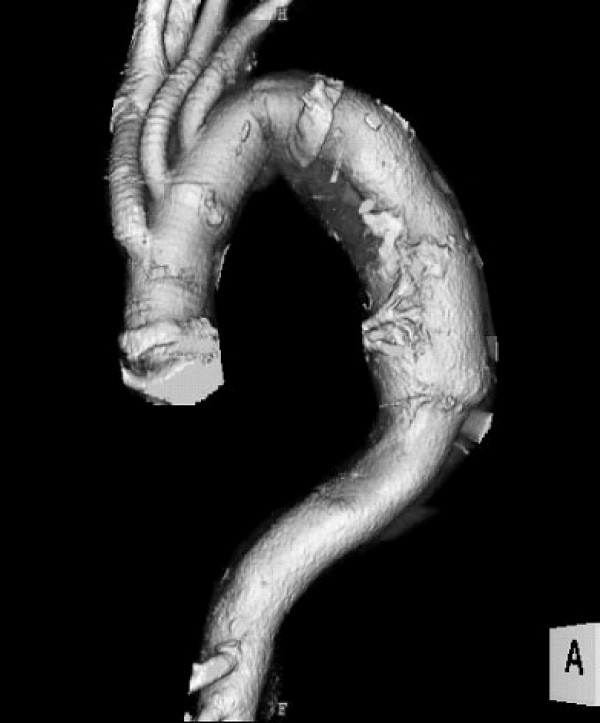
**After total aortic arch replacement with an elephant trunk procedure**.

In May 2005, extracorporeal circulation was established, then total aortic arch replacement was performed under selective cerebral perfusion. The vascular graft was a 4-branched Intergard-W (Intervascular, Flagstaff, AZ, USA). Circulation of the body was arrested at a core temperature of 26°C. An open distal anastomosis was performed with the elephant trunk procedure (diameter 24 mm, length 8 cm). After distal anastomosis, proximal anastomosis and branch arteries reconstruction were performed. Extracorporeal circulation time was 142 min, and circulatory arrest of the body time was 36 min (Figure 2).

**Figure 3:**
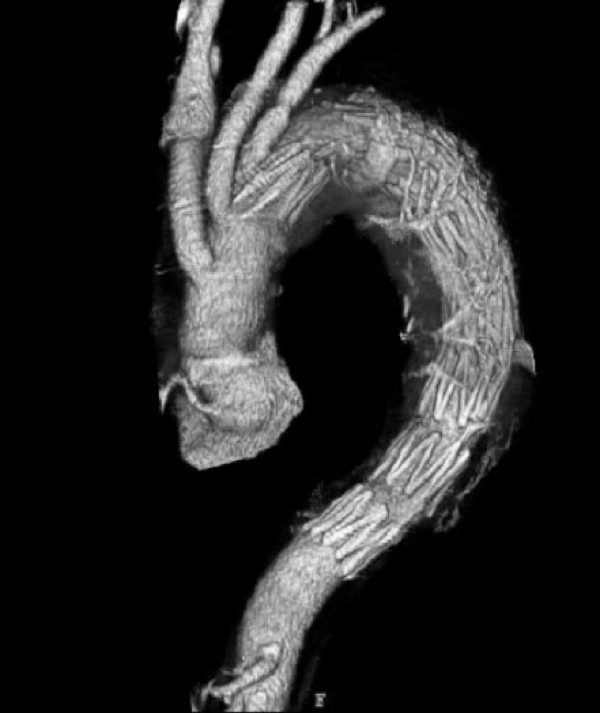
**Second-stage TEVAR using the elephant trunk graft as the proximal landing zone**.

Four weeks after arch repair, TEVAR was performed using the elephant trunk as the proximal landing zone. The stent graft was handmade by the surgical staff by connecting modified stainless steel Z stents using two support wires and was covered with an expanded polytetrafluoroethylene artificial vessel, with a window to preserve the left subclavian artery [1]. To ensure a sufficient proximal landing zone, the stent graft was placed across the distal anastomosis (Figure 3).

**Figure 4:**
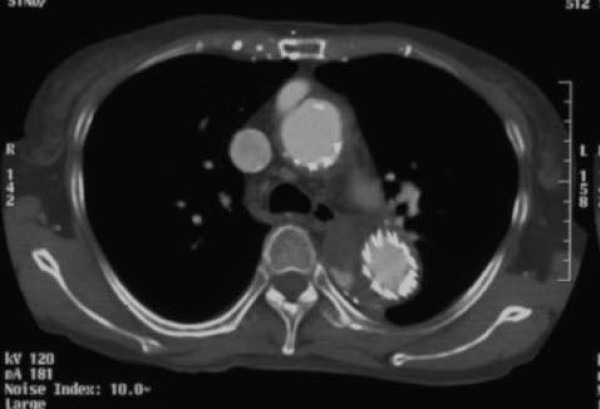
**The slight type II endoleak persists, but aneurysm dilatation has not been noted for 5 years postoperatively**.

The postoperative course was relatively uncomplicated, but a type II endoleak from intercostal artery was noted. Currently, about 5 years postoperatively, slight the type II endoleak persists, but aneurysm dilatation has not been noted, so the patient is being followed up (Figure 4).

Extensive thoracic aortic aneurysms are not uncommon in clinical practice, and the surgeon must decide whether to perform single-stage or multistage surgery. In cases with multiple adjacent aneurysms, a single-step procedure is theoretically possible, but because of excessive invasiveness, surgical outcomes may be poor [2,3]. According to a report by the Japanese Association for Thoracic Surgery, the hospital mortality rate after arch-descending aortic replacement is significantly worse than after TAR (14.3% vs. 6.5%, p < 0.001)[2]. Kouchoukos et al [3] performed single-stage repair using a clamshell incision in 46 patients and reported hospital death in only 3 patients (6.5%). However, 17% required a rethoracotomy for hemostasis, and other complications in survivors were reported, including mechanical ventilation for 72 hours or more in 42% (tracheotomy in 13%), and transient cerebral ischemia in 13%. On the other hand, when multistage surgery is selected to reduce surgical invasiveness, the surgical priority of multiple aneurysms must be decided based on diameter, morphology, and propensity for dilatation; and because of the risk of rupture during the interval between the two stages, the second stage of surgery must be scheduled as soon as possible after the first. Safi et al. [4] reported a mortality rate of 5.1% for the first stage and 6.2% for the second stage. The mortality rate during the interval between operations was 3.6%, of which 75% were the result of aneurysm rupture.

For a second-stage TEVAR after arch repair, a left thoracotomy is not necessary, so early second-stage surgery can be performed; however, there are few case reports, so long-term results are unknown. In 22 patients who underwent arch repair, Greenberg et al. [5] performed TEVAR using the elephant trunk as the proximal landing zone. They reported good mid-term results after a mean follow-up period of 17.8 months, with aneurysm-related mortality of 4.5% at 1 month, 11.3% at 1 year, and 11.3% at 2 years.

## Conclusion

The present patient still has a type II endoleak, but aneurysm dilatation has not been noted, with good long-term results. Early second-stage surgery was possible only 4 weeks after initial surgery. However, TEVAR for aortic aneurysms in Takayasu arteritis has rarely been reported [6,7], so long-term outcomes are unknown. Further careful clinical observation is therefore necessary.

## Consent

Written informed consent was obtained from the patient for publication of this case report and accompanying images. A copy of the written consent is available for review by the Editor-in-Chief of this journal.

## Competing interests

The authors declare that they have no competing interests.

## Authors' contributions

All authors read and approved the final manuscript. YO carried out the study design, Data analysis and writing, NK, NS, SK and HS performed data collection.
